# Value of detecting peri‐device leak and incomplete endothelialization by cardiac CT angiography in atrial fibrillation patients post Watchman LAAC combined with radiofrequency ablation

**DOI:** 10.1111/jce.15222

**Published:** 2021-09-01

**Authors:** Ming‐Zhe Zhao, Run‐Min Chi, Ying Yu, Qun‐Shan Wang, Jian Sun, Wei Li, Peng‐Pai Zhang, Bo Liu, Xiang‐Fei Feng, Yan Zhao, Bin‐Feng Mo, Mu Chen, Rui Zhang, Chang‐Qi Gong, Yi‐Chi Yu, Yi‐Gang Li

**Affiliations:** ^1^ Department of Cardiology Xinhua Hospital Affiliated to Shanghai Jiaotong University School of Medicine Shanghai China; ^2^ Department of Radiology Xinhua Hospital Affiliated to Shanghai Jiaotong University School of Medicine Shanghai China

**Keywords:** atrial fibrillation, endothelialization, left atrial appendage closure, peri‐device leak

## Abstract

**Objectives:**

To explore the value of detecting the peri‐device leak (PDL) and device endothelialization after left atrial appendage closure (LAAC) by cardiac computed tomography (CT) in patients with atrial fibrillation (AF), who underwent Watchman LAAC combined with radiofrequency ablation of atrial fibrillation (AFCA).

**Methods:**

Patients with symptomatic drug‐refractory atrial fibrillation at high risk of stroke (CHA_2_DS_2_‐VASc Score ≥ 2), who underwent Watchman LAAC combined with AFCA in our center from March 2017 to December 2018 were enrolled. Maximum diameter of LAA orifice was determined by preoperative CCTA. A standardized view of Watchman device was obtained by postoperative CCTA multiplannar reconstruction to evaluate the PDL and device endothelialization.

**Results:**

Approximately 84 patients post successful LAAC and AFCA were enrolled in this study. The satisfactory LAA occlusion rate was 100%. There was no death, bleeding, stroke, and device‐related thrombus (DRT) events. At 6‐month postprocedure, CCTA images evidenced complete endothelialization in 44 patients (no contrast enhancement in LAA); contrast enhancement in LAA and visible PDL in 33 patients; contrast enhancement in LAA but without PDL in seven patients (incomplete device endothelialization). Maximum diameter of LAA orifice could independently predict the occurrence of PDL (odds ratio, 1.31; 95% confidence interval, 1.11–1.55; *p* = .002), sensitivity was 69.7% and specificity was 80.4% with the cutoff value of maximum diameter of LAA orifice more than 28.2 mm on predicting PDL.

**Conclusions:**

CCTA is feasible to evaluate PDL and device endothelialization after LAAC. The maximum diameter of LAA orifice derived from CT can independently predict the occurrence of post‐LAAC PDL.

## 1 INTRODUCTION

Stroke prevention belongs to the key management among patients with atrial fibrillation (AF). Left atrial appendage (LAA) is the remnant of original left atrium (LA) in the period of embryo, and is a known major location of thrombosis in AF patients.^1^ Percutaneous endovascular left atrial appendage closure (LAAC) is increasingly performed in AF patients, especially those with contraindications to long‐term oral anticoagulants (OAC). This clinical practice conforms to the European Society of Cardiology guidelines with Class IIB recommendation for LAAC in AF patients with high‐stroke risk and contraindications to long‐term OAC.^2^

However, previous studies have revealed that postoperative peri‐device leak (PDL) might occur in more than 40% of cases post‐LAAC.^3^ Presence of PDL indicates the continued participation of LAA in the system circulation, which might still be linked with the potential risk of future stroke despite LAAC.

Recently, some researchers evidenced the presence of contrast enhancement, a sign of incomplete endothelialization of the device, post‐LAAC by cardiac computed tomography (CT).^4^ The determinants of PDL and device incomplete endothelialization post LAAC as well as the relationship between PDL and device incomplete endothelialization remain elusive now. In this study, we sought to evaluate the prevalence of PDL and the incomplete endothelialization post Watchman LAAC by the mean of cardiac computed tomography angiography (CCTA), and explore the predictors of postoperative PDL and incomplete endothelialization.

## 2 METHODS

### 2.1 Patient selection

We prospectively enrolled 84 Chinese patients with symptomatic drug‐refractory AF who underwent AFCA and LAAC between March 2017 to December 2018 in Xinhua Hospital, School of Medicine, Shanghai Jiao Tong University, China.

All patients were included with the criteria as: age more than 18 years; symptomatic nonvalvular AF refractory to antiarrhythmic drugs; and with CHA_2_DS_2_‐VASc Score more than or equal to 2 plus one of the following situations: (1) high bleeding risk (HAS‐BLED Score ≥ 3); (2) history of stroke or systemic embolic even under OAC treatment; (3) intolerance to chronic OAC because of minor bleeding caused by anticoagulation therapy; and (4) preference for LAAC device implantation as an alternative to long‐term OAC despite adequate information. The study protocol was approved by the Ethics Committee of Xinhua Hospital Affiliated to Shanghai Jiao Tong University School of Medicine. Informed consent was obtained from each patient.

### 2.2 Procedure planning

All the 84 patients underwent a series of preoperative examinations including relevant laboratory tests, 12‐lead electrocardiogram (ECG), transthoracic echocardiography (TTE), transesophageal echocardiography (TEE), and CCTA. CCTA and TEE images were routinely acquired to measure the maximum diameter of LAA orifice and exclude LA or LAA thrombus.

### 2.3 Procedure

Catheter ablation of AF (AFCA) and LAAC were performed via femoral venous access under local anesthesia, and heparin was used to achieve a target activated coagulation time of more than 250 s. Every patient accepted AFCA firstly and then followed with Watchman LAAC closure.

Under conscious sedation, a decapolar catheter was positioned in the coronary sinus and two transseptal accesses were obtained through right femoral vein. Mapping and ablation were performed under the guidance of CARTO (Biosense Webster) or Ensite (St. Jude Medical) 3‐dimensional electroanatomic mapping systems in addition to standard fluoroscopy. For patients with paroxysmal AF, standard pulmonary vein isolation (PVI) was performed and for those with persistent AF, additional linear and/or complex fragmented atrial electrogram ablations were performed according to the physician's discretion. Sinus rhythm was restored by either ablation or electric cardioversion.

LAAC procedure was under local anesthesia. The orifice diameter and depth of the LAA were measured by TEE before procedure. TEE was introduced under deep sedation to reconfirm the position of the device before release. A mean left atrial pressure above 10 mmHg was obtained before measurement. Transeptal puncture was done by standard fluoroscopy, and the X‐ray images were taken in right anterior oblique (RAO) 45°. Only WATCHMANTM 2.5 (Boston Scientific) devices were used. The device with appropriate size was chosen when the depth was allowed, generally 4–6 mm larger the maximum diameter measured by TEE before procedure. The device was then advanced into the delivery sheath and deployed by sheath retraction guided by fluoroscopy. Preliminary assessment was performed by angiography and tug test under fluoroscopy to check the device position and stability. TEE was then performed to reconfirm the position with minimal (<5 mm) to no PDL. The device was released if it was verified by the assessment of PASS criteria. The specific details were described as our previous essay.^5^ Each patient received OAC therapy during follow‐up.

### 2.4 Follow‐up

All patients were required to accept follow‐up at least twice within 6 months after therapy. TEE was performed at 3 months of follow‐up to observe if satisfactory occlusion (no PDL or with PDL < 5 mm) was maintained. In case of successful occlusion, OAC was discontinued and patients were recommended for dual antiplatelet therapy (DAPT) for another 3 months followed by aspirin. Otherwise, the original OAC was continued and a repeated TEE was performed within 3 months. The second follow‐up was at 6 months including a 12‐lead ECG, Holter and CCTA. 12‐lead ECG and Holter were examined to observe the recurrence of AF, and CCTA was reapplied to evaluate postoperative PDL and device endothelialization.

### 2.5 CT imaging

Preoperative and postoperative evaluation of LA, LAA, and adjacent structures was performed by CCTA (Somatom Definition, Siemens Medical Solutions). The temporal resolution is 330 ms, the detector collimation is 64 × 0.6 mm, the tube voltage of 120 kV and tube current of 380 mA. 100 ml contrast medium was injected with 50 ml saline flush followed through elbow vein at the rate of 5 ml/s.

Image analysis was performed by the Extended Brilliance Workspace version 4.5 (Philips Healthcare). All images were analyzed by two blinded, experienced radiologists. Evaluating the consistency of the measurement results between the two radiologists, the mean values of each CT indicator were taken for follow‐up analysis. In case of disagreement in the evaluation process, the two radiologists reevaluated and discussed to reach a consensus.

**Figure 1:**
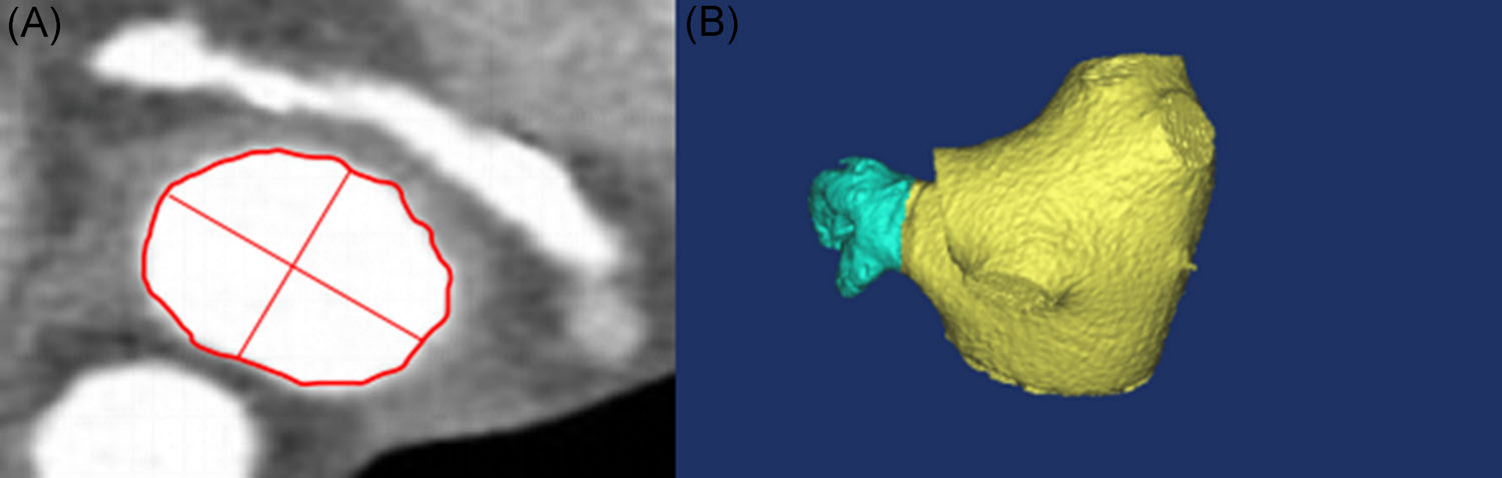
CCTA Images with multiplanar and three‐dimensional (3D) reconstruction of the LAA orifice. (A) The multiplanar reconstruction imaging of the LAA orifice, allowing to measure the maximum diameter. (B) The 3D reconstruction imaging of LA and LAA, LAA separation, LA and LAA volume calculation. CCTA, cardiac computed tomography angiography; LAA, left atrial appendage; LA, left atrium

Measurements were made of the maximum diameter of LAA orifice at the preoperative CCTA images. The double‐oblique orthogonal view of LAA orifice is obtained by the method of multiplanar imaging reconstruction (Figure 1). The orifice was defined as the plane between the circumflex artery and a point 15 ± 5 mm from the tip of the limbus, reflecting as closely as possible the site where the proximal aspect of the Watchman device would be expected to expansion.

Preoperative left atrial volume (LAV) and left atrial appendage volume (LAAV) were measured by Mimics Medical 17.0 (Materialise NV). The thinfilm cross sectional images generated by CCTA were imported into the Mimics in the DICOM data format. Images at the end‐systolic cardiac phase (when the LA cavity was largest) were selected as original data. Tissues connected to LA were then separated based on a three‐dimensional (3D) model. Separation of LA and left ventricle was bounded by the mitral valve annulus. Pulmonary veins (PV) and LAA were separated by the PV ostia and LAA orifice, respectively (Figure 1). LAV and LAAV were automatically calculated by Mimics. The specific details were described as our previous essay.^6^

PDL and device endothelialization were evaluated at the postoperative CCTA images. CT workspace was used to reconstruct the original CCTA images in 3D multiplanar, and the axial plane should be at the level of LA. For the Watchman device, first observe the position of the nitinol skeleton of the device. Afterward, move the coronal axis within the transverse window perpendicular to the coves of the parachute of the device. Afterward, align the axes on the two other viewers also perpendicular to the coves of the parachute of the device. Lastly, the center of the axes should be placed to the center of the screw‐hub. With all of the above imaging steps, the LAA occluder view for postimplantation evaluation could be established.^7^

**Figure 2:**
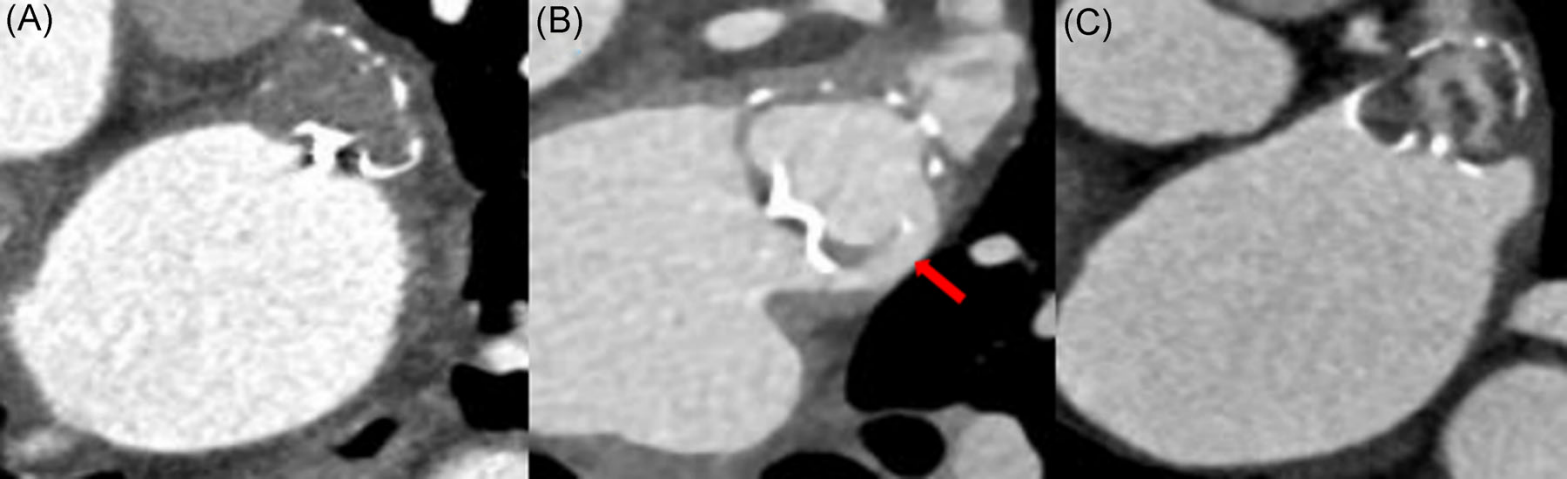
CT images showing complete LAA closure by Watchman device (A), peri‐device leak (B), and trans fabric leak (C). Panel (A) shows no evidence of contrast enhancement within the Watchman device or beyond its edges. Panel (B) shows peri‐device leak (red arrow) with contrast traversing around the device into the distal portions of the LAA. Panel (C) shows an incomplete device seal with evidence of contrast uptake within the device but not at its proximal border, suggesting residual permeability of the fabric. CT, computed tomography; LAA, left atrial appendage

Quantitative contrast assessment of LAA was carried out by measuring the average linear attenuation coefficient (Hounsfield units [HU]) in the LAA distal to the implanted device, using a circle diameter of 3 mm for the region of interest. PDL was defined as the average linear attenuation coefficient of LAA was more than 100 Hu (Figure 2), and continuous contrast enhancement was observed from LA to LAA along the side of the device.^8^ Incomplete endothelialization was defined as the average linear attenuation coefficient of LAA was more than 100 Hu (Figure 2), and continuous contrast enhancement was observed from LA to LAA through the fabric of the device (“fabric leak” from diffusion of contrast through the nonendothelialized polyethylene terephthalate membrane). The average linear attenuation coefficient less than 100 Hu was defined as LAA complete occlusion.^7^

### 2.6 Statistical analyses

Statistical analysis was performed by SPSS V22.0 (IBM Software). Measurements and indexes are expressed as mean ± *SD*. Continuous variables are described as mean ± standard deviation and were compared using Student's *t*‐test. Categorical variables are presented as percentages and were analyzed using Fisher's exact test. A receiver operating characteristic (ROC) curve was plotted to evaluate the validity and the boundary value was calculated. A *p* < .05 was considered statistically significant.

## 3 RESULTS

### 3.1 AFCA and LAA closure

A total of 84 patients were enrolled in this study. The mean age was 68.7 ± 8.0 years, including 37 females. Thirty‐eight patients were with paroxysmal AF and 46 were with persistent AF. The mean CHA_2_DS_2_‐VASc score was 3.4 ± 1.4 and the mean HAS‐BLED score was 2.2 ± 1.2. The detailed clinical characteristics are listed in Table 1.

**Table 1:** Baseline characteristics of the study population

	*N* = 84
Age, year	68.7 ± 8.0
Female	37 (44.0%)
BMI, kg/m^2^	24.9 ± 3.2
Paroxysmal AF	38 (45.2%)
CHA_2_DS_2_‐VASc score	3.4 ± 1.4
HAS‐BLED score	2.2 ± 1.2
Hypertension	54 (64.3%)
Diabetes	18 (21.4%)
Coronary artery disease	10 (11.9%)
Chronic heart failure	9 (10.7%)
Stroke or TIA	39 (46.4%)
Maximum diameter of LAA orifice, mm	
TEE	22.6 ± 3.2
CCTA	27.8 ± 5.2
Size of the Watchman device	
21 mm	3 (3.6%)
24 mm	9 (10.7%)
27 mm	37 (44.0%)
30 mm	19 (22.6%)
33 mm	16 (19.1%)

*Note:* Values presented are mean ± *SD* or *N* (%).

Abbreviations: AF, atrial fibrillation; BMI, body mass index; CCTA, cardiac computed tomography angiography; LAA, left atrial appendage; TEE, transesophageal echocardiography; TIA, transient ischemic attacks.

Mean maximum diameter of LAA orifice measured by TEE was 22.6 ± 3.2 mm, while 27.8 ± 5.2 mm by CCTA (Table 1). The paired *t*‐test showed significant differences between two groups (*p* < .001) in this study. To ensure the consistency of the examination methods, the results of maximum diameter of LAA orifice measured by CCTA were used for subsequent statistical analysis, because CCTA was used to evaluate PDL and device endothelialization in this study.

During the procedure, sinus rhythm (SR) was restored in all patients and Watchman devices were all successfully implanted. Satisfactory occlusion was achieved in 100% of patients (no PDL ≥ 5 mm). The results of five Watchman sizes used were shown in Table 1. There were no bleeding events during hospitalization and all patients were switched to OAC therapy before discharge.

At 3 months follow‐up, all the 84 patients accepted TEE, and the satisfactory occlusion rate was 100% and all patients were switched to DAPT accordingly. Neither bleeding, stroke, systemic embolic events nor device‐related thrombus (DRT) events were recorded during the mean 164‐day follow‐up. At 6 months follow‐up, all the 84 patients accepted CCTA, and 73.2% (64/84) of the patients maintained sinus rhythm. At 3 months TEE, 21 patients with PDL whose width less than 5 mm were identified, meanwhile, 33 patients with PDL whose width less than 5 mm and seven patients with trans‐fabric leak were identified by CCTA at 6 months follow‐up. The detailed TEE and CCTA results are listed in Table 2.

**Table 2:** Characterization of LAA closure results by 3‐month postoperative TEE and 6‐month postoperative CCTA

	3‐month TEE	6‐month CCTA
LAA occluded	63	44
PDL < 3 mm	19	31
PDL 3‐5 mm	2	2
PDL > 5 mm	0	0
Trans‐fabric leak	/	7
Total	84	84

Abbreviations: CCTA, cardiac computed tomography angiography; LAA, left atrial appendage; PDL, peri‐device leak; TEE, transesophageal echocardiography.

At 3 months TEE, 21 patients with PDL whose width less than 5 mm were identified, among them, PDL was detected in 18 patients (85.7%) by CCTA at 6 months follow‐up (Table 3). Totally, CCTA at 6 months detected 33 patients with PDL whose width less than 5 mm and seven patients with trans‐fabric leak, among them, PDL was detected in 18 patients (45.0%) by TEE at 3 months follow‐up, and PDL was absent in 22 patients (55.0%) at 3 months postprocedure as detected by TEE (Table 4).

**Table 3:** CCTA results detected by 6‐month follow‐up for the patients with PDL detected by 3‐month postoperative TEE

Number	Sex	Age	3‐month TEE	6‐month CCTA	Number	Sex	Age	3‐month TEE	6‐month CCTA
1	F	81	PDL	No PDL	12	M	51	PDL	PDL
2	F	75	PDL	PDL	13	M	75	PDL	No PDL
3	F	64	PDL	PDL	14	M	69	PDL	PDL
4	M	71	PDL	PDL	15	M	70	PDL	PDL
5	M	79	PDL	PDL	16	M	67	PDL	PDL
6	F	75	PDL	PDL	17	M	79	PDL	PDL
7	M	68	PDL	PDL	18	M	77	PDL	PDL
8	M	67	PDL	PDL	19	F	82	PDL	PDL
9	M	67	PDL	PDL	20	F	58	PDL	PDL
10	F	66	PDL	PDL	21	M	56	PDL	PDL
11	F	77	PDL	No PDL					

Abbreviations: CCTA, cardiac computed tomography angiography; PDL, peri‐device leak; TEE, transesophageal echocardiography.

**Table 4:** TEE results detected by 3‐month follow‐up for the patients with PDL and trans‐fabric leak detected by 6‐month postoperative CCTA

Number	Sex	Age	3‐month TEE	6‐month CCTA	Number	Sex	Age	3‐month TEE	6‐month CCTA
1	M	55	No PDL	PDL	21	M	70	PDL	PDL
2	M	66	No PDL	PDL	22	M	67	PDL	PDL
3	F	75	PDL	PDL	23	M	71	No PDL	PDL
4	F	64	PDL	PDL	24	M	79	PDL	PDL
5	M	69	No PDL	PDL	25	M	61	No PDL	PDL
6	M	71	PDL	PDL	26	M	77	PDL	PDL
7	M	79	PDL	PDL	27	M	62	No PDL	PDL
8	F	65	No PDL	PDL	28	F	76	No PDL	PDL
9	F	75	PDL	PDL	29	F	82	PDL	PDL
10	M	68	PDL	PDL	30	F	58	PDL	PDL
11	M	67	PDL	PDL	31	F	78	No PDL	PDL
12	M	67	PDL	PDL	32	M	56	PDL	PDL
13	F	60	No PDL	PDL	33	M	61	No PDL	PDL
14	M	63	No PDL	PDL	34	F	73	No PDL	Trans‐fabric leak
15	F	66	PDL	PDL	35	F	61	No PDL	Trans‐fabric leak
16	F	80	No PDL	PDL	36	F	85	No PDL	Trans‐fabric leak
17	M	51	PDL	PDL	37	F	67	No PDL	Trans‐fabric leak
18	M	69	PDL	PDL	38	M	73	No PDL	Trans‐fabric leak
19	M	65	No PDL	PDL	39	F	57	No PDL	Trans‐fabric leak
20	M	61	No PDL	PDL	40	F	62	No PDL	Trans‐fabric leak

Abbreviations: CCTA, cardiac computed tomography angiography; PDL, peri‐device leak; TEE, transesophageal echocardiography.

### 3.2 Peri‐device leak

In 51 cases (60.7%) there was no PDL detectable by CCTA. A leak with a width of less than 3 mm was present in 31 cases (36.9%), and a leak width of 3–4.9 mm was present in two cases (2.4%). None were found to have a severe leak more than 5 mm in width. Baseline characteristics according to the presence or absence of PDL are listed in Table 5.

**Table 5:** Comparison between patients with and without peri‐device leak

	All (*N* = 84)	No PDL (*N* = 51)	PDL (*N* = 33)	*p* value
Age, year	68.7 ± 8.0	69.3 ± 8.3	67.8 ± 7.8	.464
Female	37 (44.0%)	26 (51.0%)	11 (33.3%)	.123
BMI, kg/m^2^	24.9 ± 3.2	25.0 ± 3.3	24.8 ± 3.2	.760
Hypertension	54 (64.3%)	32 (62.7%)	22 (66.7%)	.817
Diabetes	18 (21.4%)	10 (19.6%)	8 (24.2%)	.786
Paroxysmal AF	38 (45.2%)	26 (51.0%)	12 (36.4%)	.262
NT‐proBNP (pg/ml)	836.2 ± 929.1	746.7 ± 829.2	974.7 ± 1076.6	.418
Troponin (ng/ml)	0.03 ± 0.14	0.02 ± 0.03	0.06 ± 0.23	.015
Fibrinogen (g/L)	2.88 ± 0.58	2.85 ± 0.58	2.93 ± 0.60	.818
d‐dimer (mg/L)	0.42 ± 2.25	0.55 ± 2.88	0.22 ± 0.45	.223
LVEF (%)	63.5 ± 6.2	64.2 ± 4.6	62.5 ± 8.1	.077
Maximum diameter of LAA orifice (mm)	27.8 ± 5.2	25.8 ± 3.7	30.9 ± 5.7	.009
Preoperative LAAV (ml)	8.73 ± 4.54	7.91 ± 3.31	10.59 ± 5.37	.006
Preoperative LAV (ml)	143.4 ± 46.2	137.3 ± 43.0	152.9 ± 50.9	.429
Watchman device size (mm)	28.3 ± 3.1	27.5 ± 3.1	29.5 ± 2.8	.877
Compression rate	19.9 ± 6.3	21.2 ± 6.9	18.2 ± 5.2	.398

*Note:* No PDL, no visible peri‐device leak group; PDL, visible peri‐device leak group.

Abbreviations: AF, atrial fibrillation; ALT, alanine transaminase; BMI, body mass index; LAA, left atrial appendage; LAAV, left atrial appendage volume; LAV, left atrial volume; LVEF, left ventricular ejection fraction; PDL, peri‐device leak; Scr, serum creatinine.

Preoperative troponin, maximum diameter of LAA orifice, and LAAV differed between with and without PDL groups, while age, sex, body mass index (BMI), presence of hypertension/diabetes, AF pattern, preoperative NT‐ProBNP, fibrinogen, d‐dimer, LVEF, LAV, or device diameter did not. On univariate logistic regression analysis, predictors of PDL were maximum diameter of LAA orifice, preoperative LAAV and device size, while age, sex, BMI, presence of hypertension/diabetes, AF pattern, NT‐ProBNP, troponin, fibrinogen, d‐dimer, LVEF, or LAV were not (Table 6).

**Table 6:** Univariate logistic regression analysis

	*B*	Wald	OR (95% CI)	*p* value
Age	−0.024	0.709	0.977 (0.925–1.032)	.400
Female	−0.732	2.497	0.481 (0.194–1.193)	.114
BMI	−0.021	0.089	0.979 (0.855–1.122)	.765
Hypertension	0.172	0.134	1.187 (0.473–2.979)	.714
Diabetes	0.272	0.255	1.312 (0.457–3.766)	.614
Paroxysmal AF	−0.599	1.712	0.549 (0.224–1.347)	.191
NT‐proBNP	<0.001	1.153	1.000 (1.000–1.001)	.283
Troponin	2.788	0.800	16.240 (0.036–7306.595)	.371
Fibrinogen	0.224	0.337	1.252 (0.587–2.670)	.562
d‐dimer	−0.100	0.318	0.904 (0.638–1.282)	.573
LVEF	−0.043	1.377	0.958 (0.891–1.029)	.241
Maximum diameter of LAA orifice	0.241	14.787	1.272 (1.125–1.438)	<.001
Preoperative LAAV	0.150	5.960	1.162 (1.030–1.310)	.015
Preoperative LAV	0.008	1.913	1.008 (0.997–1.018)	.167
Watchman device size	0.221	7.253	1.248 (1.062–1.466)	.007

Abbreviations: AF, atrial fibrillation; BMI, body mass index; CI, confidence interval; LAA, left atrial appendage; LAAV, left atrial appendage volume; LAV, left atrial volume; LVEF, left ventricular ejection fraction; OR, odds ratio.

**Figure 3:**
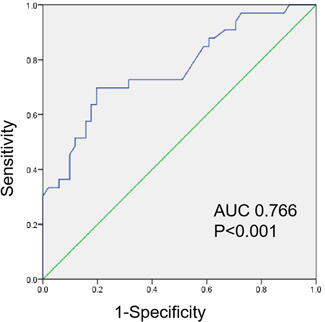
ROC curve. Receiver operating characteristic for the value of preoperative left atrial appendage orifice size measured by cardiac CT to predict the occurrence of peri‐device leak after left atrial appendage closure. AUC, area under curve; CT, computed tomography; ROC, receiver operating characteristic

Further multivariate logistic regression analysis indicated predictor of PDL was maximum diameter of LAA orifice (Table 7), which was not related with LAAV and device size. Mean maximum diameter of LAA orifice for the 84 cases was 27.8 ± 5.2 mm. Maximum diameter of LAA orifice was greater in cases with PDL compared with those without PDL (25.8 mm vs. 30.9 mm, *p* = .009). Increasing maximum diameter of LAA orifice was positively correlated with PDL (odds ratio [OR], 1.31; 95% confidence interval [CI], 1.11–1.55; *p* = .002). On ROC analysis (Figure 3), area under curve for the presence versus absence of PDL was 0.766 (95% CI: 0.659–0.872, *p* < .001). The Youden index (0.501) was the largest when maximum diameter of LAA orifice was 28.2 mm (sensitivity of 69.7% and specificity of 80.4%), suggesting that maximum diameter of LAA orifice more than 28.2 mm is more likely to demonstrate PDL.

**Table 7:** Multiple logistic regression analysis

	*B*	Wald	OR (95% CI)	*p* value
Maximum diameter of LAA orifice	0.269	9.714	1.308 (1.105‐1.549)	0.002
LAAV	0.002	0.001	1.002 (0.841‐1.194)	0.979
Device size	0.001	0.001	0.001 (0.771‐1.300)	0.993
Constant	−8.031	6.843	0.000	0.009

Abbreviations: CI, confidence interval; LAA, left atrial appendage; LAAV, left atrial appendage volume; OR, odds ratio.

### 3.3 Endothelialization

CCTA performed at 6 months postprocedure showed that the average linear attenuation coefficient of LAA was less than 100 Hu in 44 cases (52.4%), suggesting complete device endothelialization. In seven cases (8.3%) the average linear attenuation coefficient of LAA was more than 100 Hu in the absence of visible PDL, suggesting incomplete device endothelialization. Status of device endothelialization could not be determined in those patients with PDL by CCTA (*n* = 33).

Age, sex, BMI, presence of hypertension/diabetes, AF pattern, preoperative troponin, NT‐ProBNP, fibrinogen, d‐dimer, LVEF, maximum diameter of LAA orifice, LAV, LAAV, or device size did not differ between complete and incomplete endothelialization groups. Univariate logistic regression analysis showed that all the clinical factors above were not statistically significant and could not predict the degree of device endothelialization after procedure.

## 4 DISCUSSION

The major finding of present study are as follows: 33 out of 84 AF patients, who were underwent Watchman LAAC combined with AFCA, developed PDL, and incomplete endothelialization was found in 7 out of the 51 patients without PDL by CCTA at 6 months post procedure. Maximum diameter of LAA orifice could be used to predict the occurrence of PDL. These data thus indicate the close association between LAA orifice size and the occurrence of PDL post LAAC.

### 4.1 CCTA and LAAC

PDL is a common phenomenon post LAAC. TEE used to be the most common used method for the preoperative and postoperative evaluation of LAAC, including the detection of postprocedure PDL. In recent years, studies demonstrated that CCTA could be a promising alternative to TEE on the preoperative and postoperative evaluation of LAAC.^9^ The high spatial resolution and multiplanar reconstruction capability of CCTA can therefore effectively evaluate the relevant preoperative indicators, and the postoperative complications post LAAC.

CCTA examination before LAAC can assist the 3D electrophysiological mapping system to construct the LA model during catheter ablation.^10^ In addition, preoperative CCTA can measure the maximum diameter of LAA orifice to select the appropriate size of the Watchman device. In our study, the preoperative TEE defined average value of the maximum diameter of LAA orifice was 22.6 ± 3.2 mm, while the value defined by CCTA was 27.8 ± 5.2 mm (*p* < .001), suggesting that the maximum diameter of LAA orifice measured by TEE was underestimated. Previous study also showed comparable measurement results of LAA orifice derived either form CCTA with DSA or intracardiac echocardiography, while there was poor consistence on measurement results of LAA orifice derived from TEE and other modalities.^11^ In line with the previous report,^12^ smaller LAA orifice was also demonstrated in our study. Above results thus suggest that there is a potential risk to select a smaller LAAC device, if the choice was made surely on preoperative TEE measurement, which might evidently increase the risk of postoperative PDL. Thus, preoperative CCTA should be integrated into the preoperative algorithm to reduce the risk of PDL.

In our study, postoperative CCTA was done to evaluate the presence of PDL and device endothelialization at 6 months post LAAC. Previous study found that the detection efficacy on detecting post‐LAAC PDL was higher by CCTA than by TEE (51.0% vs. 34.3%, *p* = .016).^13^

### 4.2 Maximum diameter of LAA orifice and PDL

Imprecise assessment of LAA orifice, for example, the selection of too small LAAC device size based on TEE measurement, at preoperative phase is one reason for the occurrence of PDL after the procedure. Other factors linked to PDL are also suggested.^14^ LAA used to be considered to possess enough compliance to accommodate a larger size LAAC device. However, the remodeling of LA and LAA in AF patients may result in reduced compliance of LA and LAA, particularly in response to a compressible LAAC device that is deployed at relatively low radial force. Histological studies showed that the LAA specimens of AF patients existed obvious dilatation, stretching and reduction of pectinate muscle volume.^15^ In addition, most patients with chronic AF will exhibit significant thickening of endocardium and deranged thickening elastic fibers extending to the epicardium.^16^ All these changes will reduce the elasticity and compliance of LAA orifice, which increases the risk of postoperative PDL due to mismatch of LAAC device and LAA orifice.

The maximum diameter of LAA orifice and LAA volume are indirect evaluation indexes of LA remodeling. In this study, there were significant differences in the maximum diameter of LAA orifice (*p* = .009) and LAA volume (*p* = .006) between the PDL group and no PDL group, suggesting that the remodeling and reduced compliance of LAA were related to the presence of PDL. Furthermore, in the multivariate logistic regression analysis, the maximum diameter of LAA orifice served as the only significant indicator, which independently predicted the risk of PDL after LAAC procedure (OR, 1.31; 95% CI, 1.11–1.55; *p* = .002). The probability of postoperative PDL is 1.31 times higher with an increase of the maximum diameter of LAA orifice by 1 mm.

For those patients with larger size of LAA orifice and higher risk of PDL, the application of Watchman device may be suboptimal and relate to incomplete LAAC, since the available maximum diameter of the Watchman LAAC device is 33 mm now. Larger Watchman LAAC device is therefore warranted in the future to cover the need of these patients.

#### 4.2.1 Incomplete endothelialization after LAAC

CCTA can not only display PDL but also incomplete endothelialization at the distal site of LAA by signs of contrast enhancement post device implantation, while TEE is not available.

Previous report showed that satisfactory endothelialization could be achieved within 45 days after LAAC device implantation in the canine model.^17^ However, several clinical observation studies found that delayed endothelialization or incomplete endothelialization of LAAC device might occur in some patients, which was linked with increased risk of DRT, stroke, and systemic embolic events.^18^

In our study, CCTA performed at 6 months post‐LAAC showed that among 51 patients without PDL, complete device endothelialization was achieved in 44 patients (86.3%), while there were seven patients with incomplete device endothelialization (13.7%). This result is in line with a previous study, which reported incomplete endothelialization in 7 out of 79 patients (8.9%) at 3 months post LAAC with Watchman device.^13^

Totally in our study, PDL detected at 3 months by TEE was persistent at 6 month, as detected by CCTA postprocedure in the majority of patients (85.7%). And CCTA at 6 months detected 33 patients with PDL whose width less than 5 mm and 7 patients with trans‐fabric leak, among them, 18 patients (45.0%) were identified by 3 months TEE, and 22 patients (55.0%) were not. The clinical implication of PDL and incomplete endothelialization defined by CCTA in our patient remains to be determined during the planned long‐term follow‐up. It sounds that both PDL and incomplete endothelialization do not affect short‐term outcome of patients post LAAC, since adverse events were similar and rare postprocedure between patients with or without PDL and with or without incomplete endothelialization during the 6 months follow‐up post LAAC and AFCA.

Our patients will undergo long‐term follow‐up to see if the complete endothelialization could be achieved or not in the seven patients with incomplete endothelialization, and to compare the outcome of patients among various groups. Prospective long‐term follow‐up studies with large patient cohort are definitively needed to validate the value of CTTA on defining post LAAC PDL and incomplete endothelialization, CCTA derived maximum diameter of LAA orifice on predicting post LAAC PDL, and the clinical implication on outcome including the incidence of stroke, DRT or systemic embolic events of PDL and incomplete endothelialization post LAAC. Moreover, future studies should also evaluate if the maximum diameter of LAA orifice could be used as a screening index for AF patients before LAAC on risk stratification of post LAAC PDL and incomplete endothelialization and to see if this index could be integrated to other parameters to establish a compressive coding system for individualized decision making regarding the postoperative anticoagulation program.

### 4.3 Limitation

In our patient cohort, there were no patients with significant PDL ≥ 5 mm, PDL > 3 mm was found in only 2.4% (2/84) patients, which is likely to limit the strength of ROC analysis. Moreover, the clinical significance of PDL is still controversial. Long term follow‐up results are controversial now on the relationship between PDL and risk of stroke and DRT.^19^ Our planned upcoming large‐scale and long‐term clinical follow‐up and prospective control study are helpful to answer this question.

## 5 CONCLUSION

CCTA is feasible to evaluate PDL and device endothelialization after LAAC. The maximum diameter of LAA orifice can independently predict the occurrence of postoperative PDL of LAAC. Future studies are warranted to validate the value of maximum diameter of LAA orifice as a screening index for judgment of PDL risk in patients before LAAC, and as a reference basis for the decision making of individualized anticoagulant therapy after LAAC and AFCA.

## Data Availability

The data that support the findings of this study are available from the corresponding author upon reasonable request.
